# Application of AI in the identification of gastrointestinal stromal tumors: a comprehensive analysis based on pathological, radiological, and genetic variation features

**DOI:** 10.3389/fgene.2025.1555744

**Published:** 2025-07-04

**Authors:** Lei Zheng, Dan-Wen Jin, Hong-Wei Yu, Ze Yu, Li-Yong Qian

**Affiliations:** ^1^ Department of Pathology, Zhoushan Hospital, Wenzhou Medical University, Zhoushan, Zhejiang, China; ^2^ Department of General Surgery, Zhoushan Hospital, Wenzhou Medical University, Zhoushan, Zhejiang, China; ^3^ The Laboratory of Cytobiology and Molecular Biology, Zhoushan Hospital, Wenzhou Medical University, Zhoushan, Zhejiang, China

**Keywords:** artificial intelligence, gastrointestinal stromal tumors, pathological analysis, radiological features, genetic variation

## Abstract

Gastrointestinal stromal tumors (GISTs) are uncommon tumors that arise from the gastrointestinal tract, making their early diagnosis and precise identification essential for the effective clinical management. Recently, the use of artificial intelligence (AI) technologies in medicine has grown significantly, showcasing remarkable potential, especially in analyzing the pathological images, radiological features, and genetic variations. This work compiles the most recent research on the application of AI in detecting and identifying GISTs, focusing on its role in pathological image analysis, the extraction of radiological characteristics, and the interpretation of genomic data. By offering a thorough overview of these advancements, this article aims to provide a valuable reference for future research and clinical practices related to the diagnosis and treatment of GISTs.

## 1 Introduction

**FIGURE 1:**
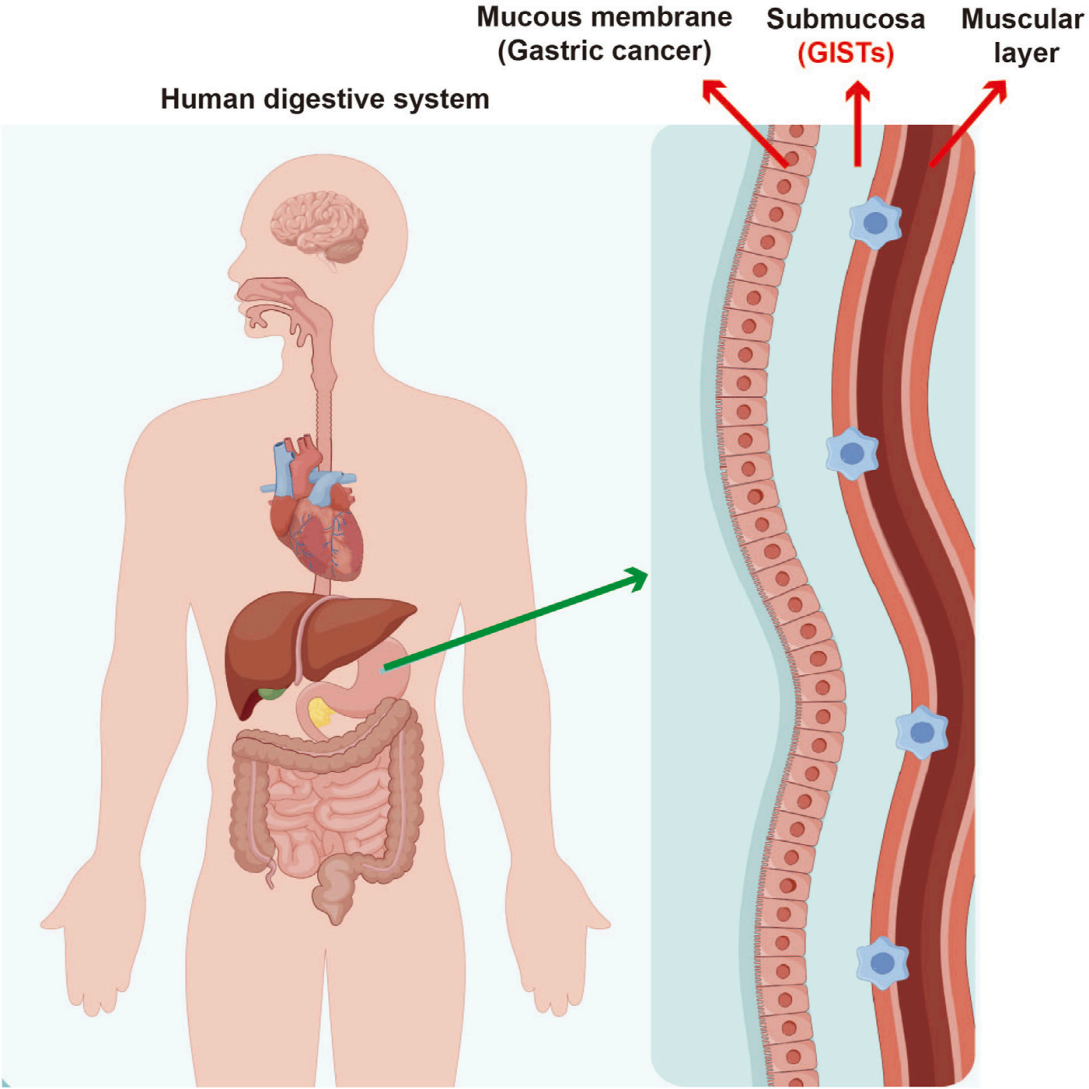
The common site of gastrointestinal stromal tumors.

Gastrointestinal stromal tumors (GISTs) are a distinct type of mesenchymal tumor that primarily originate from the interstitial cells of Cajal found in gastrointestinal tract (Figure 1). These tumors have attracted considerable clinical interest because of their potential to become malignant and their diverse presentations, which make diagnosis and management quite complex. Historically, GISTs have posed significant challenges in detection and differentiation from other subepithelial lesions (SELs), highlighting the need for advanced diagnostic techniques to achieve accurate identification and develop suitable treatment plans (Yang et al., 2020). Early recognition of GISTs is vital, as timely intervention can greatly affect patient outcomes; these tumors have potential to metastasize if not addressed promptly. Therefore, it is essential for healthcare providers to have a thorough understanding of the pathology and clinical implications associated with GISTs to ensure effective diagnosis and treatment.

Conventional diagnostic methods for identifying GISTs include endoscopic ultrasound (EUS), various imaging techniques, and histopathological evaluations. While these methods have been the standard approach, they come with certain limitations, such as subjective interpretation and variability in diagnostic accuracy. For example, although EUS can be a useful tool in the diagnosing GISTs, its effectiveness can be compromised by the operator’s level of experience and the challenges associated with distinguishing GISTs from other soft tissue tumors (Zhang B. et al., 2023). Imaging modalities like computed tomography (CT) can sometimes lack necessary specificity, resulting in false positives or negatives that may mislead clinical management. These limitations highlight the urgent need for enhanced diagnostic tools that can improve both the accuracy and efficiency of detecting GISTs.

In recent years, artificial intelligence (AI) has become a transformative force in the medical field, providing innovative solutions to long-standing diagnostic challenges. It’s application in gastrointestinal pathology, especially in the detection and diagnosis of GISTs, has demonstrated promising outcomes. The AI algorithms are capable of analyzing large volumes of imaging and histopathological data, which allows for more objective assessments and minimizes the potential for the human error (Lu et al., 2023). The capability of AI to combine various types of data, such as genetic information and imaging characteristics, can significantly enhance our understanding of GISTs. This integration may lead to improved risk assessment and tailored treatment strategies for patients. As AI technology becomes increasingly integrated into clinical settings, it has the potential to transform the way GISTs are diagnosed, making the process more accurate and efficient, which in turn could lead to better outcomes for patients (Hirai et al., 2022). The following sections will explore the advancements in AI applications for detection of GISTs, emphasizing the integration of pathological, radiological, and genetic data. This comprehensive approach aims to enhance diagnostic accuracy and improve patient outcomes by leveraging the strengths of each data type.

## 2 Applications of AI in pathology

The integration of AI into pathology has brought about significant changes, particularly in improving the accuracy and efficiency of diagnoses. AI technologies, especially deep learning algorithms, are now being employed to analyze large volumes of digital pathology images, which helps pathologists make better-informed decisions. Digital pathology refers to the process of converting traditional glass slides into digital formats, resulting in whole slide images (WSIs) that can be examined using AI. These technological advancements not only aid in diagnosing a variety of medical conditions but also enhance ability to predict patient outcomes based on pathological characteristics. As AI technology continues to advance, its role in pathology is anticipated to grow, ultimately leading to better patient care and more efficient workflows in clinical environments (Berezowska et al., 2023; Go, 2022). For instance, some researchers have proposed the RRCART model based on deep learning to analyze the causes of identification errors in frozen lymph node sections of breast cancer (such as section quality and missed diagnosis of micrometastasis), providing methodological references for prevention and control of pathological misdiagnosis of GIST (Zhao et al., 2024). Furthermore, through comparing performance of AI and manual analysis in the classification of colorectal cancer tissues, it was confirmed that the convolutional neural network (CNN) achieved an accuracy rate of 97% in tumor-normal tissue classification, highlighting the potential of AI in automatic classification of pathological images (Doğan and Yılmaz, 2024).

### 2.1 Acquisition and processing of digital pathology images

The acquisition and processing of digital pathology images play a vital role in integrating AI into the field of pathology. The WSI technology is utilized to capture high-resolution images of tissue samples, which can subsequently be analyzed via various computational methods. However, the substantial size and complexity of these images present considerable challenges for efficient processing. Promising advancements have been made with AI algorithms, especially those utilizing deep learning, which have demonstrated their ability to automate the analysis of these images. This automation facilitates tasks such as segmentation, feature extraction, classification, achieving high levels of accuracy. In addition, techniques like generative adversarial networks (GANs) are being applied to enhance image quality, normalize staining, and ultimately improve the overall analysis of pathology images (Jose et al., 2021; Salvi et al., 2020). The efficient processing and analysis of these images play a key role not only in aiding diagnosis but also in supporting the research initiatives that seek to understand disease mechanisms and develop targeted therapies.

### 2.2 Application of deep learning algorithms in GIST pathology identification

**FIGURE 2:**
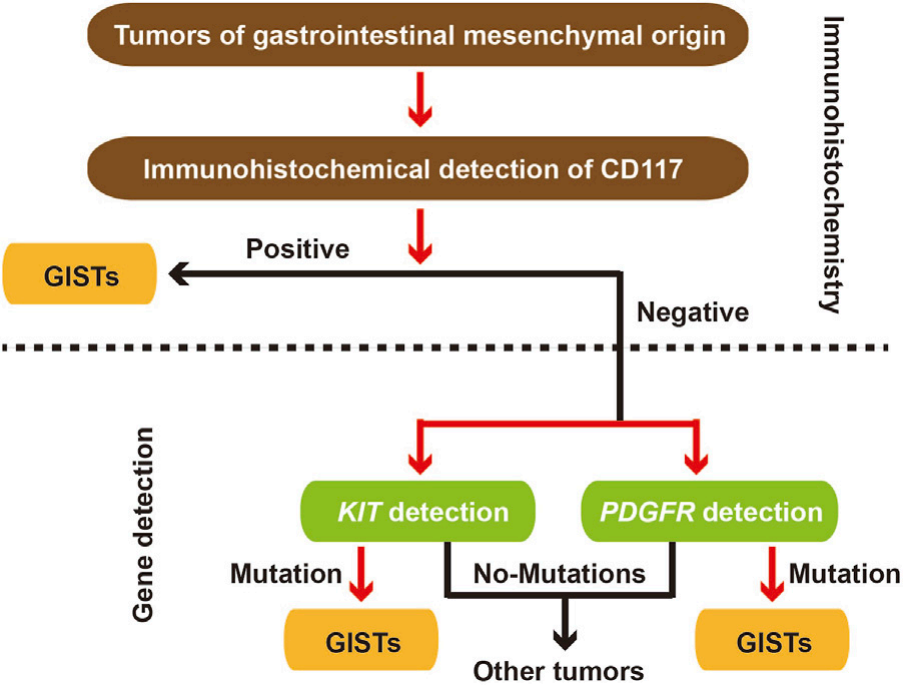
Routine detection of gastrointestinal stromal tumors.

GIST is a common stromal tumor, and its pathology and gene mutation detection are very important for identification of GIST (Figure 2). Deep learning algorithms are being increasingly utilized for the identification of GISTs in pathology. These algorithms take advantage of extensive datasets composed of EUS images to develop models that can effectively differentiate GISTs from other subepithelial lesions. For example, research has shown that artificial intelligence models can reliably assess potential malignancy of GISTs by analyzing EUS images, resulting in impressive sensitivity and specificity rates (Lu et al., 2023; Minoda et al., 2022). The application of convolutional neural networks (CNNs) has proven to be highly effective in the automated detection and classification of GISTs. This technology significantly diminishes the dependence on subjective interpretations typically made by pathologists. Besides, AI-assisted diagnostic tools have demonstrated their potential to enhance the accuracy of risk stratification for GIST patients diagnosed. This improvement in risk assessment can ultimately inform treatment decisions, leading to better patient outcomes (Zhang B. et al., 2023; Seven et al., 2022).

### 2.3 Correlation analysis between pathological features and clinical prognosis

The relationship between pathological features identified through AI analysis and clinical prognosis represents a vital research area in pathology. Through combining insights derived from AI with clinical data, researchers can create predictive models that evaluate likelihood of disease progression and patient outcomes. For instance, study has demonstrated that certain pathological characteristics, when examined using the AI algorithms, are associated with survival rates and treatment responses across different types of cancer, including GISTs (Yang et al., 2020; Narimisaei et al., 2024). The correlations identified in this research not only deepen our understanding of tumor biology but also pave the way for personalized treatment strategies. By predicting clinical outcomes based on specific pathological features, we highlight the transformative potential of artificial intelligence in patient management within oncology. This advancement could lead to more effective therapeutic and targeted approaches, ultimately improving patient care and outcomes (Gomes et al., 2023; Zhang F. et al., 2023).

## 3 The role of radiomics in GIST detection

Radiomics is an emerging area in medical imaging that focuses on extracting a wide range of quantitative features from medical images, which can significantly improve diagnostic accuracy and guide treatment decisions. This approach is particularly promising for GISTs, the most common type of mesenchymal tumors that can be malignant. By utilizing radiomics, healthcare professionals can gain detailed insights into tumor characteristics that are not visible to the naked eye, enabling more accurate risk assessment and tailored treatment planning. Recent research has underscored the effectiveness of various imaging techniques, such as CT and EUS, in diagnosing and managing GISTs. These imaging methods not only help in identifying GISTs but also allow for the evaluation of their biological behavior and potential malignancy through advanced image analysis techniques, including AI (Yang et al., 2020; Minoda et al., 2022).

### 3.1 Basic concepts and methods of radiomics

Radiomics is a technique that extracts a wide range of features from medical images, which can be grouped into categories such as shape, intensity, texture, and wavelet features. These features are quantified through algorithms that analyze the pixel values in the images, helping to uncover patterns that may be linked to specific disease characteristics. The typical workflow involves several steps: first, acquiring the images; next, segmenting the region of interest; then extracting the relevant features; and finally, analyzing the data using statistical or machine learning methods. In context of GIST detection, radiomics can significantly improve diagnostic process via providing objective data that helps distinguish between benign and malignant lesions. Moreover, the incorporation of AI into radiomics enhances its capabilities through automating the feature extraction process and facilitating the creation of predictive models. These models can support clinicians in making well-informed decisions about patient management (Zhang B. et al., 2023; Lu et al., 2023).

### 3.2 Feature extraction and AI model construction

The extraction of imaging features plays a crucial role in the radiomics workflow, especially for GISTs. These features are obtained from various imaging techniques, such as CT and EUS, and they offer valuable insights into the tumor’s shape, texture, and overall variability. AI models, particularly those that employ machine learning methods, can be trained using these features to forecast important outcomes, including the likelihood of tumor malignancy, the response to treatment, and overall prognosis for patients. For example, research has shown that AI-enhanced EUS can achieve impressive sensitivity and specificity when differentiating GISTs from other subepithelial tumors, highlighting the ability of AI models to improve the diagnostic precision. Furthermore, the creation of risk stratification models based on radiomic features can assist in making informed therapeutic decisions, enabling personalized treatment strategies that take into account the unique risk profiles of individual patients (Lu et al., 2023; Minoda et al., 2022).

### 3.3 Comparative studies of radiomics and traditional imaging

Comparative studies between radiomics and traditional imaging techniques have highlighted the benefits of integrating quantitative imaging features into the diagnostic process for GISTs. While traditional imaging methods are valuable, they often depend on subjective interpretation, which can overlook subtle differences in tumor characteristics. In contrast, radiomics offers a more objective and thorough analysis of image, resulting in enhanced diagnostic performance. For instance, artificial intelligence-assisted EUS has demonstrated greater accuracy compared to conventional endoscopic assessments, showcasing the potential of radiomics to better differentiate GISTs from other lesions. Additionally, research has shown that radiomic models can surpass traditional imaging metrics in predicting tumor behavior and treatment outcomes, indicating that the incorporation of radiomics into clinical practice could significantly enhance the management of GISTs (Minoda et al., 2022; Peng et al., 2023). In addition, Neuro-radiology has emerged as foremost discipline conducting studies on AI. Nonetheless, this innovative technology is being effectively evaluated in various other operational environments, including surgical procedures and radiation therapy. Within this framework, AI has been demonstrated to substantially decrease resource expenditure and costs while upholding a high standard of quality. Besides, pathological diagnosis and the advancement of novel systemic treatments represent two more domains where AI has exhibited encouraging preliminary findings (Di Nunno et al., 2022).

## 4 AI analysis of genetic variation characteristics

### 4.1 Overview of genetic variations associated with GIST-related genes

GISTs are mainly caused by mutations in specific genes, particularly *c-KIT* and *PDGFRA* genes, which play a crucial role in their development. Around 85% of GISTs have mutations in the *c-KIT* gene, while a smaller fraction shows mutations in *PDGFRA*. Wild-type GISTs, which do not have mutations in these common genes, make up about 5%–10% of cases and pose unique challenges for diagnosis and treatment due to their varied characteristics. Recent research has broadened the understanding of genetic differences in GISTs, uncovering new fusion genes that are involved in tumor formation and resistance to treatment, especially in cases resistant to imatinib. Whole-exome sequencing (WES) has shown varying oncogenic mutations in high-grade GISTs, emphasizing the intricate nature of genetic changes that can affect how tumors behave and respond to therapies. Furthermore, the discovery of mutations in genes like *NF1* and *BRAF* in certain instances highlights the diverse genetic makeup of GISTs, which can inform targeted treatment approaches and enhance patient outcomes (Cho et al., 2020; Qian et al., 2022).

The relationship between genetic variations in GISTs and their behavior is a dynamic area of research that holds significant implications for prognosis and treatment strategies. Studies have shown that specific mutations, particularly in *c-KIT* and *PDGFRA* genes, correlate with distinct clinical outcomes and tumor characteristics. For instance, GISTs harboring mutations in c-KIT exon 11 typically respond better to imatinib therapy than those with mutations in other regions or those that are wild-type. Additionally, the presence of further mutations in genes such as *NF1* and *BRAF* may suggest a more aggressive tumor phenotype and a poorer prognosis. Recent studies have also examined how genetic alterations contribute to the progression from low-grade to high-grade tumors, underscoring the necessity of understanding the genetic landscape for accurate predictions of tumor behavior. This research underscores the potential for personalized treatment approaches tailored to the genetic profile of GISTs, paving the way for more effective therapeutic interventions (Yang et al., 2020; Minoda et al., 2022).

### 4.2 Application of AI in genomic data interpretation

AI has become a powerful tool in analyzing genomic data, especially regarding GISTs. AI algorithms, particularly those based on machine learning, are being designed to improve how we interpret the complex genomic information produced by next-generation sequencing (NGS). These models help identify pathogenic variants and assess their clinical relevance, which is crucial for advancing precision medicine. For example, AI systems can sift through extensive datasets to find patterns and relationships that might not be obvious with traditional analytical methods. AI has demonstrated potential in enhancing diagnostic accuracy and risk assessment for GISTs, proving to be an invaluable resource in clinical decision-making via combining clinical and genomic data. Additionally, AI-driven methods can simplify the variant interpretation process, cutting down time and expertise needed for manual analysis and allowing for quicker identification of actionable mutations (Lu et al., 2023; Huang et al., 2022). Studies have shown combining proteomic and transcriptomic characteristics, including genes involved in tumorigenic pathways, can effectively identify patients with a reduced possibility of ICIs response to immunotherapy for metastatic melanoma (Mallardo et al., 2024).

The main contributions of AI methods to the interpretation of genomic data:

1. Handling massive and complex data: AI algorithms (especially machine learning) can efficiently process and analyze the huge and complex genomic data generated by next-generation sequencing (NGS), which is beyond the capabilities of traditional methods.
2. Identifying pathogenic variations: AI can “mine” data to discover potential pathogenic genetic variations and assess their clinical significance, which is conducive to precise diagnosis of diseases.
3. Revealing Hidden patterns: AI excels at identifying subtle patterns, associations, and trends (such as interactions between genes or associations with environmental factors) in large datasets. These patterns are not easily detectable by conventional analytical methods and may contain significant biological or clinical insights.
4. Enhance risk assessment and prediction: By integrating and analyzing genomic data and clinical information, AI can assist in more precise disease risk prediction, providing stronger support for individualized treatment decisions.

Therefore, we believe that through its powerful data processing capabilities and pattern recognition capabilities, when analyzing the vast genomic information, AI not only improves the accuracy and depth of discovering pathogenic variations and their clinical significance, but also greatly simplifies the process and increases the speed, thereby promoting application of precision medicine in clinical practice. Especially in the management of diseases like GISTs that rely on genomic information.

## 5 AI comprehensive application: integration of pathological, imaging, and genetic data

### 5.1 Necessity of multimodal data fusion

The necessity for multimodal data fusion in healthcare is highlighted through the growing complexity of medical conditions and the demand for more comprehensive diagnostic tools. Traditional diagnostic approaches often depend on a single type of data, which can result in incomplete evaluations and potential misdiagnoses. For instance, GISTs can be particularly difficult to distinguish from other subepithelial lesions when relying solely on imaging techniques. However, by integrating imaging data with genetic information and histopathological findings, the accuracy of diagnoses can be greatly enhanced. Research indicates that AI-assisted endoscopic ultrasonography (EUS) can achieve impressive sensitivity and specificity in diagnosing GISTs when it is combined with clinical and genetic data (Hirai et al., 2022; Minoda et al., 2022). The integration of multimodal data fusion methodologies significantly improves diagnostic performance while also aiding in risk stratification and treatment planning. This advancement is essential for contemporary healthcare systems, as it allows for a more comprehensive understanding of patient conditions and facilitates more informed decision-making in clinical settings.

### 5.2 Comprehensive performance evaluation of AI models

Evaluation of AI models in the context of multimodal data fusion is essential for confirming their reliability and effectiveness in clinical settings. A thorough performance assessment should include a range of metrics, such as sensitivity, specificity, positive predictive value (PPV), and negative predictive value (NPV). For example, AI models designed for diagnosing GISTs have shown impressive accuracy rates, with combined sensitivity and specificity values suggesting their potential as dependable diagnostic tools (Lu et al., 2023; Zhan et al., 2023). Evaluating AI models across a variety of clinical scenarios, including different imaging techniques and diverse patient demographics, is crucial for determining their generalizability and reliability. Systematic reviews and meta-analyses allow researchers to compile data from numerous studies, offering a more comprehensive understanding of an AI model’s effectiveness in various contexts. This aggregated information can significantly aid clinicians in making informed decisions regarding the integration of these technologies into their practice (Gomes et al., 2023; Joo et al., 2024).

### 5.3 Construction of clinical decision support systems

Development of clinical decision support systems (CDSS) that utilize integrated multimodal data represents a significant advancement in optimizing clinical workflows and enhancing patient care. These systems can aid healthcare providers by consolidating complex information from diverse sources and delivering evidence-based recommendations that are customized for the individual patients. The incorporation of AI into CDSS has demonstrated potential in enhancing diagnostic accuracy and alleviating the cognitive burden on clinicians, especially in high-pressure settings like emergency departments (Rout et al., 2024; van Baalen et al., 2021).

AI-driven CDSS have the potential to revolutionize patient care via analyzing imaging data in conjunction with patient history and genomic information, thereby suggesting personalized treatment options for conditions such as cancer. However, the successful implementation of these systems is not without its challenges. The key issues include ensuring user acceptance among healthcare professionals, achieving interoperability with existing electronic health records, and providing ongoing training and support staff. Healthcare organizations can significantly enhance the effectiveness of CDSS, through addressing these critical areas, ultimately leading to improved patient outcomes (García-Rudolph et al., 2024; Stagg et al., 2021).

## 6 Future prospects and challenges

### 6.1 Potential development directions of AI technology in GIST research

The future of AI technology in GIST research is set to experience remarkable progress, especially in diagnostic imaging and predictive analytics. Recent studies have underscored the promise of AI-assisted imaging techniques, such as endoscopic ultrasound (EUS), in enhancing diagnostic accuracy and distinguishing GISTs from other subepithelial lesions. As AI algorithms evolve, they are expected to utilize advanced deep learning methods to interpret intricate imaging data, which will improve the accuracy of GIST characterization. Additionally, combining AI with genomic data could pave the way for personalized treatment approaches tailored to individual tumor profiles, leading to more effective and less harmful targeted therapies. The creation of AI-driven risk stratification models may also empower clinicians to forecast the chances of malignant transformation in GISTs, thereby informing surveillance and intervention strategies. Nevertheless, realizing these advancements will require a collaborative effort among researchers, clinicians, and technologists to ensure that AI tools remain clinically relevant and meet the highest standards of safety and efficacy.

### 6.2 Data privacy and ethical issues

As AI technologies increasingly integrate into healthcare, especially within the realm of GIST research, data privacy and ethical considerations are becoming significant challenges. The implementation of AI typically requires the analysis of extensive patient data, which brings forth concerns regarding confidentiality and security of sensitive health information. Ethical dilemmas can surface from the potential misuse of this data, particularly when it is shared with third parties or utilized for commercial purposes without obtaining patient consent (Chiruvella and Guddati, 2021). Growing dependence on AI systems brings with it significant risks associated with algorithmic bias, particularly when models are trained on datasets that do not accurately represent the diversity of patient population. Such biases can result in unequal diagnosis and treatment outcomes, which can adversely affect patient care.

To mitigate these risks, it is crucial to develop strong frameworks for data governance that not only safeguard patient privacy but also promote research and innovation. This involves adopting rigorous data de-identification methods, obtaining informed consent from patients, and maintaining transparency regarding the development and application of AI systems. We can work towards ensuring that AI technologies are both effective and equitable in their application within healthcare by prioritizing these measures (Jwa and Poldrack, 2022). Ongoing dialogue among stakeholders - such as patients, healthcare providers, and policymakers - will be essential for effectively navigating the ethical landscape of AI in GIST research.

### 6.3 Barriers and solutions in clinical application

Clinical application of AI technologies in managing GISTs encounters various barriers that need to be overcome to ensure effective integration into healthcare practices. A major challenge is the regulatory landscape, which frequently fails to keep pace with technological advancements. This discrepancy leads to uncertainty surrounding the approval and utilization of AI tools in clinical environments (Ma et al., 2024). There is a pressing need for standardized protocols and interoperability among different AI systems to facilitate their smooth integration into current workflows. The intricate nature of AI algorithms can pose challenges for healthcare professionals, many of whom may not have the training required to effectively interpret insights generated by AI. To address these challenges, it is essential to invest in education and training programs that empower clinicians with skills necessary to confidently use AI tools. Additionally, promoting collaboration between AI developers and clinical practitioners can result in the development of user-friendly interfaces that improve the clarity and usability of AI outputs (Varghese, 2020). Establishing clear guidelines for ethical use of AI in clinical practice, along with ongoing monitoring of AI systems for their performance and potential biases, is crucial for enhancing patient care and preserving trust in the healthcare system.

## 7 Expectation

Given that there are relatively few reports on the application of artificial intelligence in the identification of gastrointestinal stromal tumors (GIST), this study references the application of AI in other tumors to guide future development of AI applications in GIST, exploring 3 dimensions: pathology, imaging, and genetic mutations.

### 7.1 Pathological image analysis

Based on the RRCART model of deep learning, analyze the causes of identification errors in frozen lymph node sections of breast cancer (such as section quality and missed diagnosis of micrometastases), provide methodological references for prevention and control of pathological misdiagnosis of GIST (Zhao et al., 2024).

By comparing performance of AI and manual analysis in the classification of colorectal cancer tissues, it was confirmed the convolutional neural network (CNN) achieved an accuracy rate of 97% in tumor-normal tissue classification, highlighting the potential of AI in the automatic classification of pathological images (Doğan and Yılmaz, 2024).

### 7.2 Radiomics and radiological characteristics

Explore application of AI in preclinical tumor imaging, emphasizing that deep learning can accelerate the development of targeted imaging agents and provide new ideas for discovery of imaging markers for GIST (Küper et al., 2023).

Review the progress of AI in multimodal image integration of bladder cancer (such as MRI/CT feature mining), and prove that it can improve the repeatability of diagnosis and is applicable to the feature extraction of GIST images (Yang et al., 2025).

### 7.3 Genomic variations and multi-omics integration

Systematically summarize the role of AI in precise cancer treatment, including target prediction, immunotherapy response, and identification of long non-coding RNAs, providing a framework for the analysis of variations in GIST driver genes (such as KIT/PDGFRA) (Wang et al., 2025).

Propose multi-omics (genome + transcriptome + microbiome) combined with AI to predict the response to immunotherapy in non-small cell lung cancer, laying a methodological foundation for the study of the association between GIST gene variations and treatment (Mei et al., 2024).

### 7.4 Multimodal data fusion and comprehensive management

Utilizing spatial transcriptomics and AI to analyze the microenvironment of pancreatic cancer, achieving cell interactions and biomarker mining, which can be transferred to pathology-gene integration analysis of GIST (Maurya et al., 2024).

Review the application of machine learning models (such as SVM, random forest) in mesothelioma risk prediction, and prove that multi-model collaboration can improve diagnostic accuracy (Ram et al., 2025).

Furthermore, we believe application goals and endpoint indicators of AI in GIST recognition can be discussed in the following dimensions:

1. Pathological image analysis:

Objective: Distinguish GIST from other subepithelial lesions through EUS images; Predict the malignant potential of GIST (low, medium, high risk), and assist in clinical decision-making (Pallio et al., 2023).

Indicator: Improve diagnostic performance and classification accuracy.

2. Radiomics feature extraction:

Objective: Quantify texture features using CT/MRI images to replace invasive examinations; evaluate the response to targeted therapy and predict the risk of recurrence (Barat et al., 2024).

Indicator: Screen out the imaging markers related to c-KIT mutations and output the progression-free survival through the radiomics model.

3. Analysis of Genetic Variations:

Objective: Correlate imaging features with specific mutations to guide the selection of targeted drugs.

Indicator: Improve the detection sensitivity of AI models for high-risk mutations.

## 8 Conclusion

In recent years, the integration of AI into the detection and diagnosis of GIST has become a significant advancement in medical diagnostics. Through combining AI with traditional diagnostic methods, the reliability of results is enhanced, and the workflow in clinical settings is streamlined, which ultimately leads to improved patient outcomes.

The significance of multidisciplinary collaboration is paramount in context of implementing AI technologies for GIST diagnosis. Successful integration of these technologies relies on the cooperation of a diverse group of stakeholders, including oncologists, pathologists, radiologists, and data scientists. This teamwork promotes a holistic approach to research, merging clinical insights with sophisticated computational methods.

In summary, the future of AI in detection and diagnosis of GIST appears promising; however, its successful integration into clinical practice hinges on continuous collaboration, thorough research, and a steadfast commitment to ethical standards. By nurturing these components, we can fully harness the potential of AI, ultimately enhancing diagnostic capabilities and improving patient care in the field of GIST.
